# Transcriptome sequencing and differential expression analysis of natural and BTH-treated wound healing in potato tubers (*Solanum tuberosum* L.)

**DOI:** 10.1186/s12864-022-08480-1

**Published:** 2022-04-05

**Authors:** Hong Jiang, Xue Li, Li Ma, Yingyue Ren, Yang Bi, Dov Prusky

**Affiliations:** 1 College of Horticulture, Gansu Agricultural University, Lanzhou, 730070 People’s Republic of China; 2 College of Food Science and Engineering, Gansu Agricultural University, Lanzhou, 730070 People’s Republic of China; 3 Department of Postharvest Science, Agricultural Research Organization, 7505101 Rishon LeZion, Israel

**Keywords:** Potato tubers, Wound healing, BTH, Transcriptome, Metabolic mechanism

## Abstract

**Background:**

Wound healing is a representative phenomenon of potato tubers subjected to mechanical injuries. Our previous results found that benzo-(1,2,3)-thiadiazole-7-carbothioic acid S-methyl ester (BTH) promoted the wound healing of potato tubers. However, the molecular mechanism related to inducible wound healing remains unknown.

**Results:**

Transcriptomic evaluation of healing tissues from potato tubers at three stages, namely, 0 d (nonhealing), 5 d (wounded tubers healed for 5 d) and 5 d (BTH-treated tubers healed for 5 d) using RNA-Seq and differentially expressed genes (DEGs) analysis showed that more than 515 million high-quality reads were generated and a total of 7665 DEGs were enriched, and 16 of these DEGs were selected by qRT-PCR analysis to further confirm the RNA sequencing data. Gene ontology (GO) enrichment analysis indicated that the most highly DEGs were involved in metabolic and cellular processes, and KEGG enrichment analysis indicated that a large number of DEGs were associated with plant hormones, starch and sugar metabolism, fatty acid metabolism, phenylpropanoid biosynthesis and terpenoid skeleton biosynthesis. Furthermore, a few candidate transcription factors, including MYB, NAC and WRKY, and genes related to Ca^2+^-mediated signal transduction were also found to be differentially expressed during wound healing. Most of these enriched DEGs were upregulated after BTH treatment.

**Conclusion:**

This comparative expression profile provided useful resources for studies of the molecular mechanism via these promising candidates involved in natural or elicitor-induced wound healing in potato tubers.

**Supplementary Information:**

The online version contains supplementary material available at 10.1186/s12864-022-08480-1.

## Background

Wound healing of potato tubers is an event mediated by wound-related signals, which leads to cell regeneration and deposition at wound sites to form the biopolymer suberin [1, 2]. The main function of suberin deposition is to seal off the injured tissue, and wound-induced suberin production provides protection from water evaporation and resistance to pathogen infection by acting as a physical barrier [3, 4]. The formation of this physicochemical barrier signifies that complex structural and physiological progress is completed and established on wounded tubers. In general, during long-term storage, potato tubers require wound healing to reduce respiration and decay [5]. However, a process of natural healing without any measures takes a longer time. Therefore, some new strategies for rapid wound healing need to be developed to reduce the postharvest quality deterioration of potato tubers.

Benzo-(1,2,3)-thiadiazole-7-carbothioic acid S-methyl ester (BTH), an analogue of salicylic acid (SA), has been reported to induce systemic acquired resistance (SAR) to protect against pathogen infection [6, 7]. The exogenous application of BTH induced disease resistance in some fruits and vegetables, including apples [8], oranges [9], pitaya [10] and potato [11, 12]. Our previous results showed that BTH-inducible wound healing restricts weight loss and pathogen attack of potato tubers by increasing suberin and lignin deposition at wound sites [13], which was attributed to the elevation of phenylpropanoid metabolism during wound healing. Moreover, BTH application promoting wound healing was also documented to be related to the involvement of reactive oxygen species (ROS) metabolism [14], and it was considered to act as a signalling molecule and cross-link phenylpropanoid-derived monomers to the cell wall during wound healing. Similarly, our recent results found that BTH could promote the wound healing of muskmelons by activating phenylpropanoid metabolism and stimulating ROS production [15].

Regarding the regulatory mechanisms of BTH treatment, the protein profile has been reported during ripening of muskmelon fruit, revealing a series of defence and stress response proteins [16]. In terms of the mechanism of wound healing on potato tubers, metabolite profiling revealed a broader range of suberin-associated compounds, including organic acids, sugars, amino acids, phenylpropanoids and aliphatics, that contributed the most to wound healing of potato tubers [17]. Proteomic evaluation of wound healing in potato indicated the various metabolic mechanisms involved in these processes, such as cell division, cell structure, signal transduction, energy metabolism, defensive reaction and secondary metabolism [18]. Nevertheless, there are few reports on the regulatory analysis of genes participating in the biosynthetic pathways of suberin in potato tubers at the transcriptional level, especially after treatment with BTH.

Hence, we investigated the changes in gene expression profiling related to a series of metabolism such as primary metabolism of starch and sugar and secondary metabolism including fatty acid metabolism, phenylpropanoid biosynthesis, and terpenoid skeleton biosynthesis. Additionally, we also identified a large number of DEGs of plant hormones, transcription factors, and that related with Ca^2+^-mediated signal transduction triggered by natural and BTH-induced wound healing. Through the analysis, an abundance of genomic resources for potato and novel molecular insights into wound healing in potato tubers were provided.

## Results and discussion

### Overview of RNA sequencing and mapping

A total of 162.54 million (T1), 164.89 million (T2) and 188.47 million (T3) clean reads from transcriptomic libraries were generated after the processing of raw reads (Table. S1). Overall, approximately 83.98-91.86% of clean reads were mapped to the reference genome. On average, the mapped numbers (mapped ratios) from T1, T2 and T3 were uniquely 148.71 (91.51%), 142.89 (86.67%) and 159.7 million (84.69%), respectively. These reads were blasted with public databases such as GO and KEGG for further analysis.

**Fig. 1:**
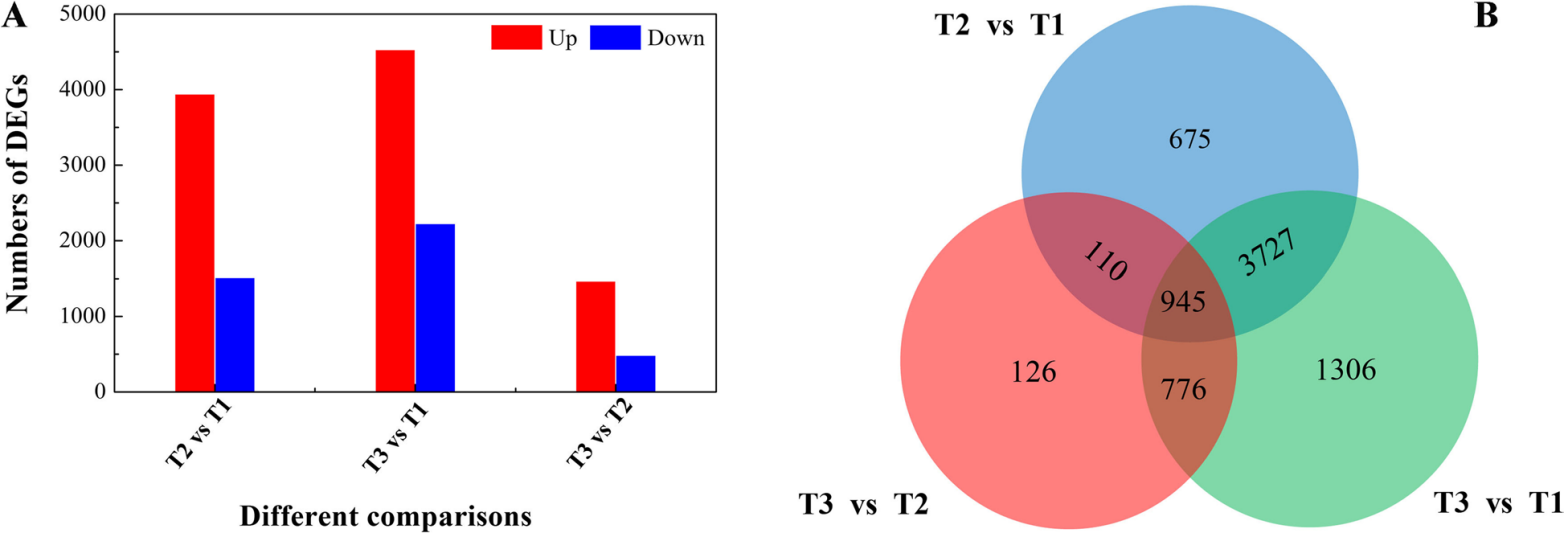
Identification and characterization of differentially expressed genes (DEGs) in different tissues. (**A**) The number of up- and down-regulated DEGs among T2 vs. T1, T3 vs. T2 and T3 vs. T1 comparisons. (**B**) Venn diagram comparison summarizing the number of differentially expressed genes among the three comparisons

Differentially expressed genes were screened by pairwise comparison using |FC| ≥ 2 and FDR <0.01 as a threshold. The results showed that the number of DEGs between T1 and T2 was 5457, including 3940 upregulated genes and 1517 downregulated genes, and a total of 1957 DEGs, including 1471 upregulated genes and 486 downregulated genes, were identified in the T3 versus T2 comparison. In addition, there were 6754 DEGs between T1 and T3, of which 4524 genes were upregulated and 2227 genes were downregulated (Fig. 1A). A Venn diagram of the DEG analysis indicated that 945 genes were shared across the three groups of paired comparisons, which accounted for 1.2% of all the DEGs. The results illustrated that 110 common genes appeared in the T2 vs. T1 and T3 vs. T2 comparisons, 776 genes were shared between T3 vs. T2 and T3 vs. T1, and 3727 genes were shared between T2 vs. T1 and T3 vs. T1 (Fig. 1B). These results indicated that most of the genes were regulated at the transcriptional level during wound healing.

### GO annotation and enrichment

GO classification was used to describe the DEG properties by biological process, molecular process and cellular component [19]. To investigate the potential functions of DEGs in the process of wound healing, a GO analysis in T2 vs. T1 comparisons was performed. A total of 4787 DEGs were classified into 30 functional categories, including 8 ‘biological process’, 8 ‘molecular functions’ and 14 ‘cellular components’, in which 3550 and 1237 genes were upregulated and downregulated, respectively, in T2 relative to T1 (Fig. S1). In each of these categories, ‘response to stimulus (9.5%)’, ‘binding (28.5%)’ and ‘nucleus (5.3%)’ were the most abundant terms, suggesting that both the upregulated and downregulated genes enriched in these categories were related to the process of wound healing.

To evaluate the functions of DEGs in tissues undergoing wound healing after treatment BTH, 25 functional categories were classified, including biological process (10), molecular functions (7) and cellular component (8) (Fig. S2). The GO analysis of 1231 upregulated and 231 downregulated genes from the T3 vs. T2 comparison showed the most significant enrichment in the biological process of ‘oxidation-reduction process’ and ‘response to stimulus’, the most significant molecular function enrichment in ‘binding’, and cellular component enrichment in ‘intergral component of membrane’.

Based on the classification of GO terms in the T3 vs. T1 comparisons, a total of 4742 DEGs, with 3450 upregulated and 1292 downregulated genes, were found to be associated with the main ontology categories (Fig. S3). In the biological process category, the largest subgroups were protein phosphorylation (3.5%) and oxidation-reduction process (7.7%). In molecular functions, the largest subgroups were transferase activity (6.7%) and binding (29.6%). In the cellular component category, the nucleus and integral component of the membrane accounted for 4.7 and 6.5%, respectively. In summary, the above GO analysis in different comparisons revealed several biological processes related to wound healing in terms of wound stress and elicitor treatment at the cellular level.

### KEGG analysis of differently expressed genes

**Fig. 2:**
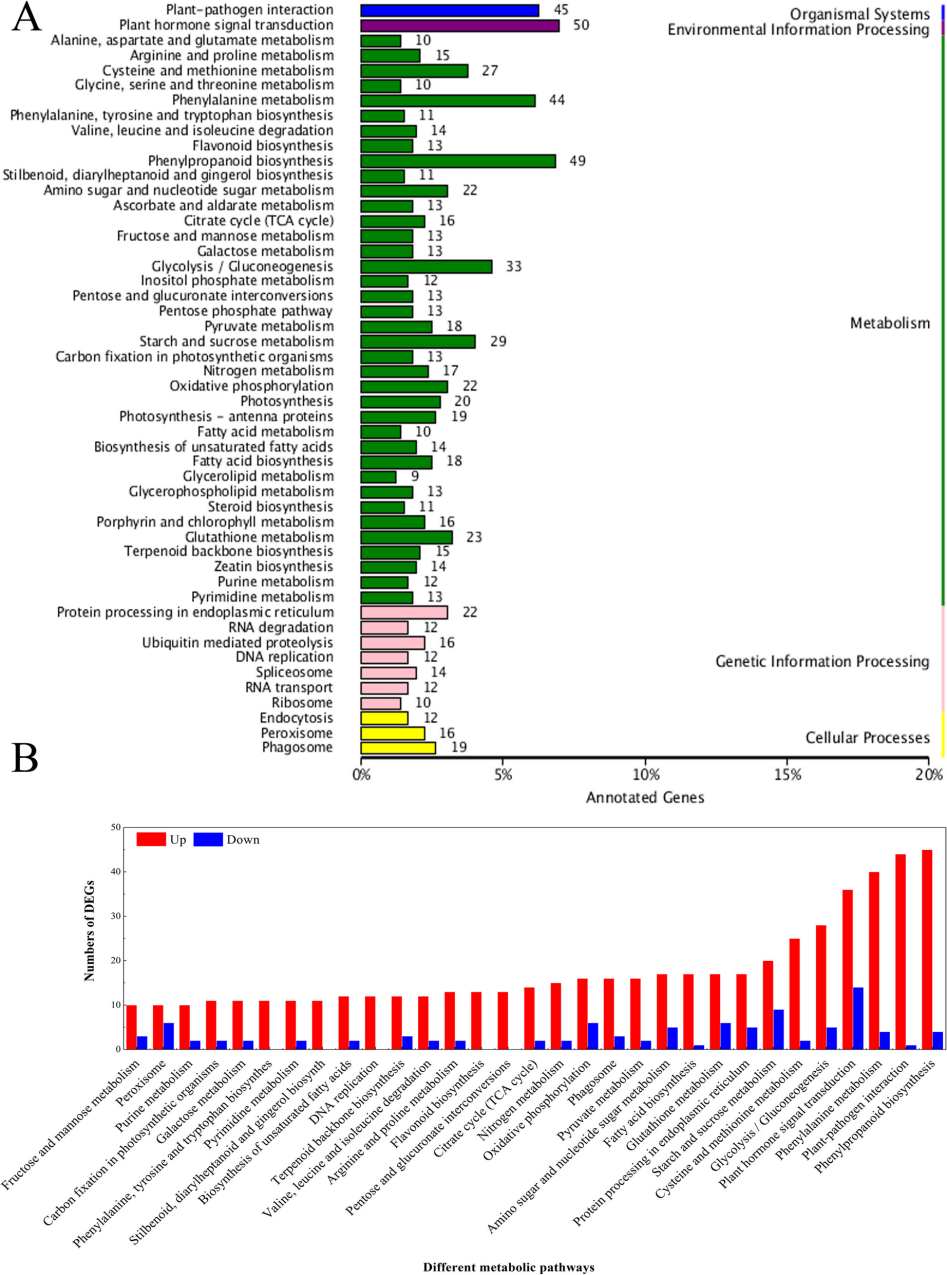
KEGG enrichment analysis of DEGs in T2 vs. T1 comparisons (**A**) and the up-regulated and down-regulated genes in selected pathways (**B**). Number of genes in each pathway is shown on y-axis; pathway categories are shown on x-axis

KEGG is a knowledge base used for systematically analysing the functions of genes. To further characterize the transcripts of DEGs affected by wounding, a KEGG pathway mapping analysis to identify functional enrichment was carried out, and the results showed that 50 pathways were assigned to different categories, including metabolism, organismal systems, genetic information, environmental information processing and cellular processes, in the transcriptomic profile comparisons of T2 vs. T1 (Fig. 2A). In the selected 31 pathways that included 652 DEGs, the upregulated and downregulated DEGs accounted for 85.1 and 14.9%, respectively (Fig. 2B). In T2 vs. T1 comparisons, genes associated with the pathways ‘plant hormone signal transduction’ ‘phenylalanine metabolism’, ‘plant-pathogen interaction’ ‘phenylpropanoid biosynthesis’ were the most enriched. These highest DEG representations in the metabolic pathways suggested that a series of specific pathways could be triggered once the potato tubers were wounded.

**Fig. 3:**
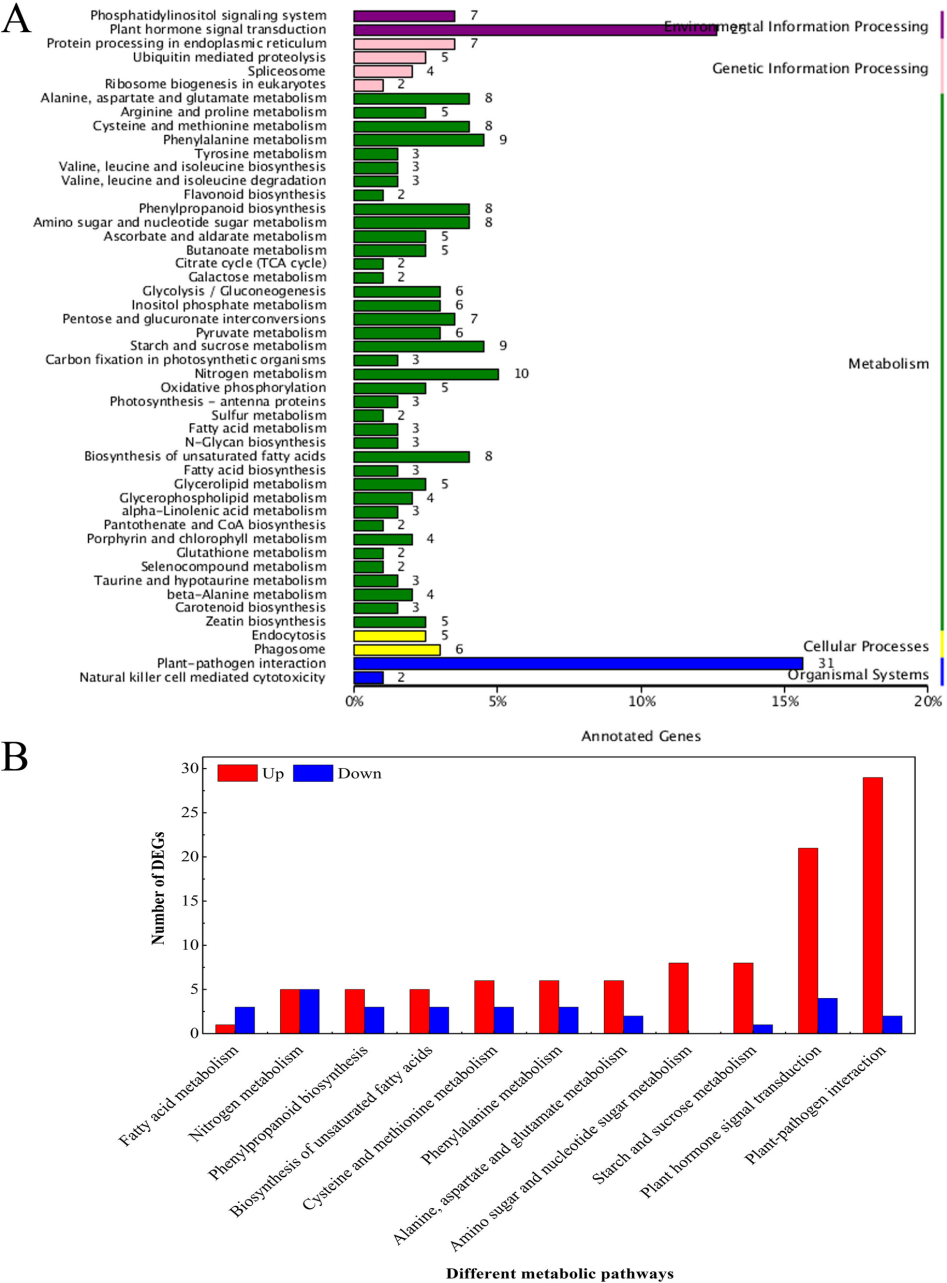
KEGG enrichment analysis of DEGs in T3 vs. T2 comparisons (**A**) and the up-regulated and down-regulated genes in selected pathways (**B**). Number of genes in each pathway is shown on y-axis; pathway categories are shown on x-axis

The KEGG analysis showed that 198 unigenes were assigned to 50 active pathways, including starch and sucrose metabolism, plant hormone signal transduction and plant-pathogen interaction, and these three pathways were the most enriched terms in the T3 vs. T2 comparisons (Fig. 3A). Moreover, out of the 50 pathways, 11 pathways with 128 DEGs were selected for further expression analysis, 100 of which were upregulated and only 28 of which were downregulated. In the plant hormone signal transduction pathway, 21 and 4 DEGs were upregulated and downregulated, while 29 and 2 DEGs were upregulated and downregulated in the plant-pathogen interaction pathway (Fig. 3B). Taken together, these DEGs were involved in related pathways regulated by BTH at the transcript level.

**Fig. 4:**
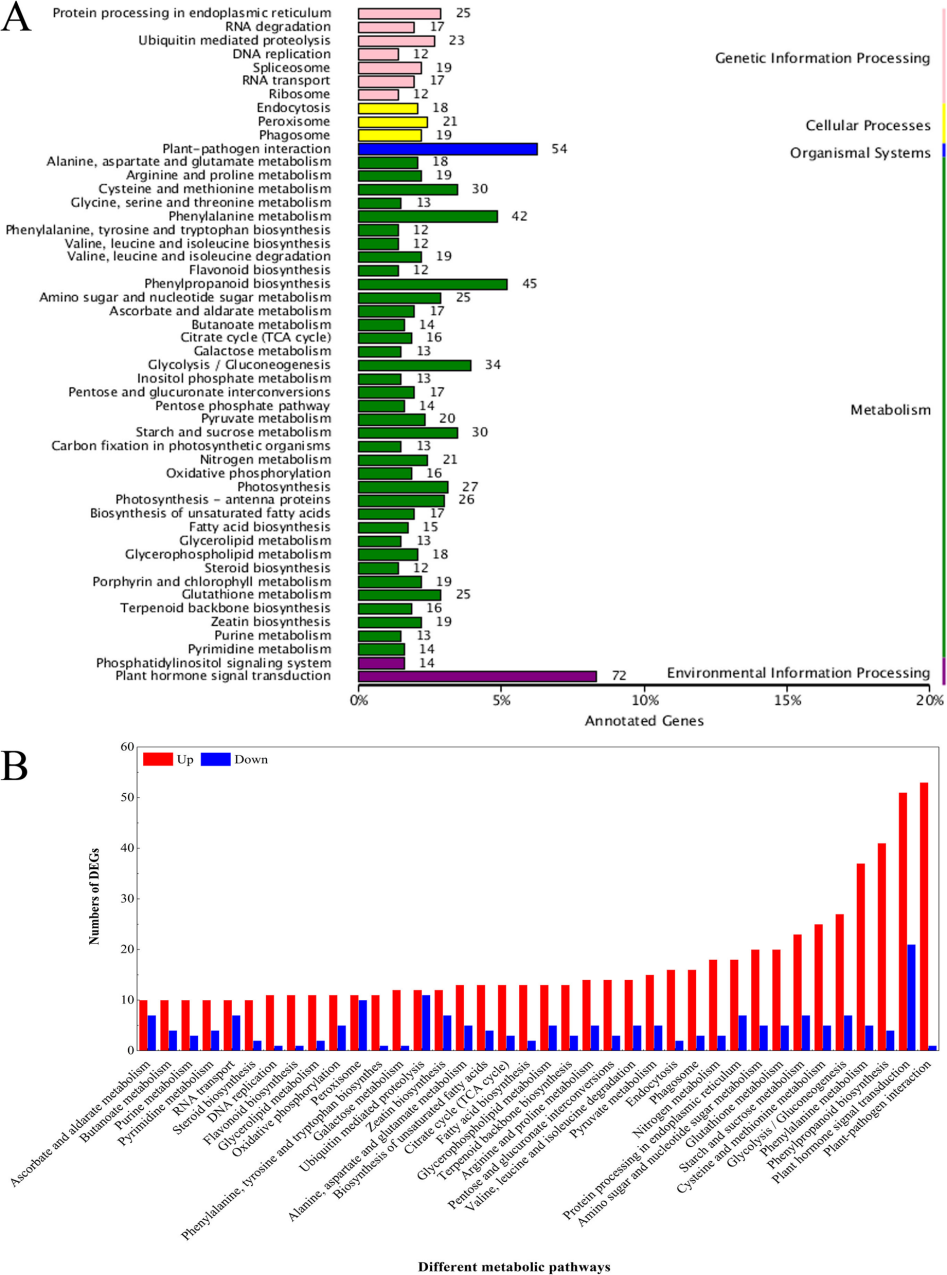
KEGG enrichment analysis of DEGs in T3 vs. T1 comparisons (A) and the up-regulated and down-regulated genes in selected pathways. Number of genes in each pathway is shown on y-axis; pathway categories are shown on x-axis

In T3 vs. T1 comparisons, 863 identified unigenes with significant matches were assigned to 5 categories, including 49 pathways (Fig. 4A). Among these categories, the unigene numbers in the phenylpropanoid biosynthesis pathway of metabolism, plant-pathogen interaction pathway of organismal systems, and plant hormone signal transduction pathway of environmental information processing were the greatest, which were 45, 54 and 72, respectively.

As shown in Fig. 4B, there were more upregulated DEGs that were significantly enriched in the selected 38 pathways than downregulated DEGs, and the plant-pathogen interaction was the pathway with the greatest number of EDGs. These results indicated that various metabolic processes occurred in response to wounding healing and that the DEGs in these pathways may play crucial roles during wound healing induced by BTH. Overall, these results highlighted a range of different molecular regulation strategies depending on the wound healing of potato tubers, and our data indicated that these candidates involved in various metabolic and biosynthetic pathways may be crucial in the response to wound healing.

### DEGs involved in major metabolic pathways

#### Phytohormone signalling

**Fig. 5:**
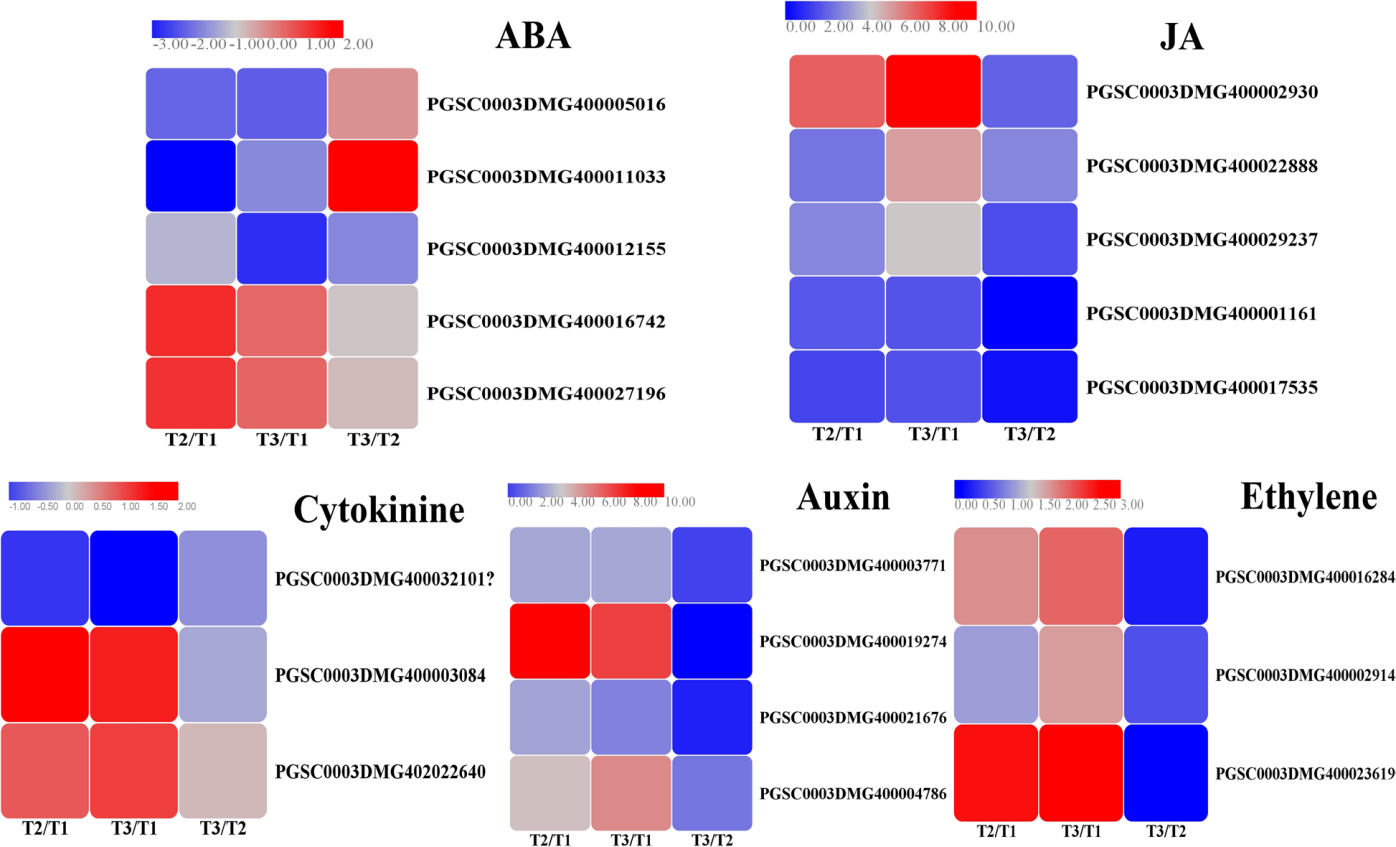
Heatmaps of DEGs involved in phytohormone signaling including abscisic acid (ABA), jasmonic acid (JA), auxin, cytokinins and ethylene. Each column represents the mean expression value (log_2_ FPKM, T2 sample are divided by T1, T3 sample are divided by T1 and T2, respectively) of three biological replicates obtained from RNA-Seq data

In plants, many stress responses, including wounding, are regulated by cross-communicating signal-transduction pathways, in which hormones such as abscisic acid (ABA), jasmonic acid (JA), auxin, cytokinins and ethylene fulfil central roles [20]. Intensive research implicated ABA as a positive or negative regulator in the activation of disease resistance or the response to wounding [21], and ABA was specifically proven to be involved in the wound healing of potato tubers [22]. In our experiment, three *PYR*/*PYL* genes of abscisic acid recpeptor were involved in the perception and transduction of ABA signals, and they were all expressed at lower levels in natural and BTH induced tissues in contrast with control (Fig. 5). however, another two *PP2C* genes were both expressed at higher levels only in natural healing tissues, instead. Therefore, the *PYP *and *PP2C* genes were considered as negative and positive regulator during wound healing. JA is a lipid-derived regulator that has been firmly implicated in the systemic plant defence response as a mobile signal, wounded plants may rapidly produce JA to initiate self-healing and regeneration reactions [23], and JA was also demonstrated to be necessary during wound periderm development that occur after 3 d of wound healing [24]. In this study, five *JAZ* genes involved in JA signalling were all significantly upregulated once tuber wound healing occurred and the upregulated fold of one gene (PGSC0003DMG400002930) was the greatest, which was 6.8-, 8.9- and 2.1- fold in T2/T1, T3/T1 and T3/T2 groups, respectively. The results suggest that ABA and JA might also play an essential role during natural and BTH-induced wound healing of potato tubers.

The repair and reinforcement of the cell wall and the activation of wound signalling pathways are typical characteristics of physiological responses to wounding and occur through hormones related to wounding, such as auxin, ethylene and cytokinine [25]. Auxin is a hormone that controls the growth and development of plants and functions in recovery from different wounds or organ loss [26]. The four auxin-responsive genes in Fig. 5 were all upregulated in natural and BTH-treated wound healing tissues, among which the *GH3* gene PGSC0003DMG400019274 was upregulated by 8.3-fold and 6.7-fold in T2/T1 and T3/T1 groups. This can be reflected by the description of indole-3-acetic acid content increased by 2- and 4-fold after 5 and 7 days of wounding [27]. Another hormone called cytokine plays diverse roles in the defence response to pathogens, including preinvasive defence, by regulating stomatal closure and postinvasive defence by inducing callose deposition or gene expression related to defence or phytoalexin generation [28]. In our study, one and two cytokinine genes were down and upregulated in natural and BTH induced tissues in contrast with non-wound tissues, respectively. Ethylene, one of the classical defence hormones, is released when plants are wounded mechanically [26]. Heyman et al. reported that the ethylene response factor (*ERF*) family coordinates stress signalling with wound healing, which initiates the reactivation of cell division [29]. In the present experiment, three *ERF* genes in the ethylene signal were all upregulated in natural and BTH-induced wound tissues compared with non-wounded tissues. And the *ERF* gene PGSC0003DMG400023619 was upregulated 2.5- and 2.6-fold in these two groups. ERF genes also involved in disease resistance, it was discovered that *CpERF7* was significantly up-regulated after BTH treatment [30]. To sum up, we speculate that the expression of these genes involved in different hormone signalling pathways was triggered by the natural or BTH-induced wound healing of potato tubers. Interestingly, the differential expression of multiple phytohormone signalling genes may indicate that there are not just linear and isolated cascades but also crosstalk reactions between these hormone signals.

#### Transcription factors

**Fig. 6:**
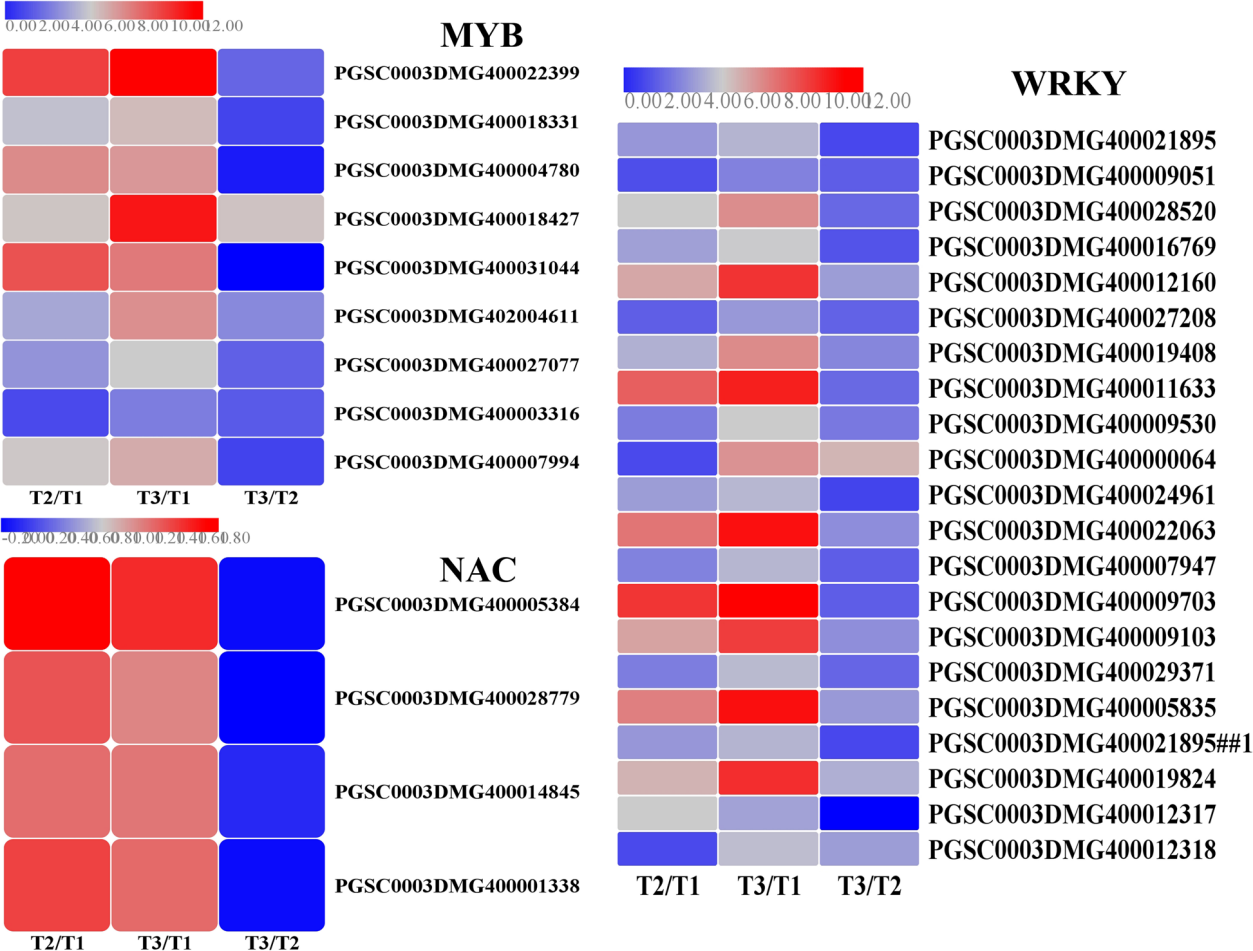
Heatmaps of DEGs encoding transcriptional factors including MYBs, NACs and WRKYs. Each column represents the mean expression value (log_2_ FPKM, T2 sample are divided by T1, T3 sample are divided by T1 and T2, respectively) of three biological replicates obtained from RNA-Seq data

Complicated transcription regulatory networks require the involvement of a series of transcription factors that belong to important constituents of signalling pathways and play pivotal roles in the response to biotic and abiotic stresses [31, 32]. These transcription factors include *MYB*, *NAC* and *WRKY*, which are encoded by a large number of genes. *MYB* transcription factors serve as one of the largest family genes and act as crucial regulators by controlling gene expression in plant development and stress responses [33]. There are 233 MYB family members that have been identified and analyzed in the potato genome and were found to confer different responses to abiotic and biotic stresses [34]. In *Arabidopsis thaliana*, the transcription levels of suberin biosynthetic genes were increased by the overexpression of *AtMYB41* and induced ferulate accumulation [35], and the transcriptional regulators *MYB58* and *MYB63 *could specifically activate lignin biosynthetic genes during secondary wall formation [36]. Lashbrooke et al. reported that *AtMYB9* and *AtMYB107* are required for suberin assembly and that the corresponding mutants led to a significant reduction in suberin monomers [37]. In kiwifruit, *AchnMYB41* and *AchnMYB107* play a positive role in the activation of *AchnFHT* (ω-hydroxyacid/fatty alcohol hydroxycinnamoyl transferase, FHT), while *AchnMYB4* works as a negative regulator, by which suberin monomer biosynthesis is therefore controlled [38]. In this study, a total of 9 potato *MYB* genes were upregulated by either natural or BTH-treated wound healing. Except for PGSC0003DMG400003316, other genes exhibited lower FPKM value below 1.0 in non-healing tissues, while they are increased more than 10 times after natural or BTH-induced wound healing (Fig. 6), it was suggested that these upregulated *MYB* genes in response to wound healing might play a regulatory role in this process.

NAC transcription factors are found in a wide range of plants and are one of the largest families and have been implicated in defensive responses to pathogens or environmental stresses [39]. In *Arabidopsis*, two *NAC* transcription factors were documented to be induced after wounding and fungal or bacterial pathogen infection [40, 41]. In potato, 110 *NAC* genes have been identified by genome-wide analysis, some of which were reported to be expressed under wounding and BTH-treated conditions [42]. In our experiment, 4 *NACs* transcription factors were all found to be upregulated in natural and BTH-treated wound healing tissues. Similar with *WRKY*, these four genes in non-healing tissues were not expressed at all, but expressed at higher level in natural and BTH-induced tissues. Especially, the gene (PGSC0003DMG400005384) showed the greatest expression level, which has been proved to repressed the suberin polyester and suberin-associated waxes deposition [43]. Therefore, we hypothesize that the upregulation of these NAC genes after wounding may regulate the process of wound healing in potato tubers.

*WRKY* transcription factors are widely expressed in various organisms and are considered as the largest family of transcription factors [44]. Many studies have shown that *WRKY* regulators defend against pathogens and provide resistance to wounding [45]. There are 79 *WRKY* family members that have been identified in the potato genome [46], 20 of which were analyzed and all of them were induced in wound healing tissues on the basis of our results. The data showed that most genes were upregulated more than 10 times. And parts of them were also significantly upregulated in BTH-treated vs. natural wound healing, for example, PGSC0003DMG400000064,PGSC0003DMG400019824,PGSC0003DMG400005835were upregulated by 5.6-, 4.0- and 3.4-fold, respectively (Fig. 6). Similarly, Chen and Chen reported that *WRKY3* and *WRKY4* from tobacco were induced by salicylic acid, which accounted for salicylic acid inducing the related kinases that could phosphorylate the WRKY protein, leading to an increase in DNA-binding activity [47]. The analysis suggested that the *WRKYs* induced by BTH may play specific roles during wound healing of potato tubers, which also provides preliminary indications of putative functions of several *WRKY* genes. However, the hypothesis related to these transcription factors requires confirmation by additional research in future studies.

#### Genes involved in Ca^2+^ signal transduction pathway

**Fig. 7:**
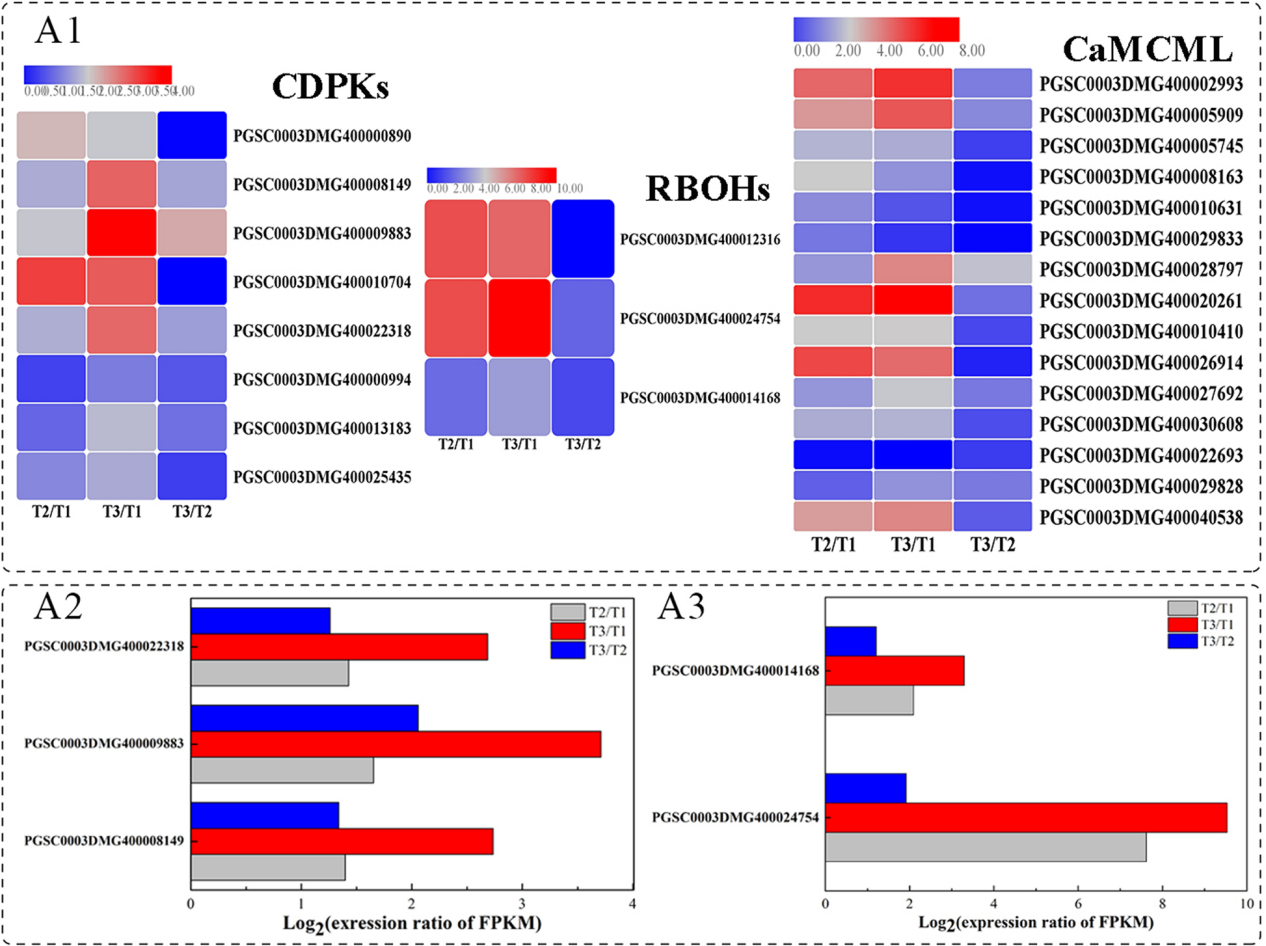
Heatmaps of DEGs encoding genes related with Ca^2+^ signal transduction during wound healing of potato tubers (A1) and the up-regulated fold of *StCDPKs* (A2) and *StRBOHs* (A3) in different comparisons. (Calcium dependent protein kinase, CDPK; Respiratory burst oxidasehomologue, RBOH; calmodulinc binding protein, CaMCaL. Each column represents the mean expression value (log_2_ FPKM, T2 sample are divided by T1, T3 sample are divided by T1 and T2, respectively) of three biological replicates obtained from RNA-Seq data)

As a second messengers, Ca^2+^ is an essential aspect of signal transduction, and it has been reported that Ca^2+^ is involved in the communication of cells and long-distance delivery [48]. The decoding and translation of information encoded by Ca^2+^ signatures is archived by Ca^2+^-binding proteins, among which calcium-dependent protein kinases (CDPKs) play particularly crucial roles. In this pathway, 8 *CDPKs* that encode key Ca^2+^-sensing proteins were significantly enriched and were induced after natural or BTH-induced wound healing occurring (Fig. 7. A1). The specific gene PGSC0003DMG400009883 was the most upregulated in each comparison, which was 1.6-, 3.7- and 2.1 fold, respectively (Fig. 7. A2). After BTH treatment, the *CDPKs* transcript level in tomato [49] or CDPK protein accumulation in banana [50] were also reported to be induced. Another calcium-binding protein, CaMCML, and a total of 15 DEGs were identified in the potato genome, which were all initiated and upregulated by wound healing except for PGSC0003DMG400022693, the upregulation fold of PGSC0003DMG400002993 and PGSC0003DMG400020261 in T2/T1 and T3/T1 reached 4-7 fold.

Respiratory burst oxidase homologues (RBOH) in plasma membranes mainly function in ROS production via signal transduction pathways [51]. *Rbohs*, a multigene family, serve as multispanning transmembrane proteins in the Ca^2+^ signal transduction pathway and require Ca^2+^ binding for their activation in response to environmental stresses [52]. It has been reported that early ROS production after potato wounding participates in the signal cascade reaction, and later ROS are thought to be tied to suberin poly (phenolic) cross-linking in wound healing [53, 54]. In our transcriptomic data, three Rbohs genes were identified in healing tissues, while two of them (PGSC0003DMG400024754 and PGSC0003DMG400014168) exhibited higher expression levels in healing tissues, the former showed the greatest upregulation fold, especially under the action of BTH application (Fig. 7. A3). Thus, the expression of these proteins reflects that the Ca^2+^-mediated signal transduction pathway via related proteins may be involved in the natural or BTH-induced healing process.

#### Starch and sugar metabolism

Suberization is a complex process including a set of physiological and metabolic events that require the coordinated regulation of genes involving primary and secondary pathways both spatially and temporally [55]. The analysis of KEGG enrichment highlighted some functional categories: DEGs associated with starch and sugar metabolism, fatty acid metabolism, phenylpropanoid biosynthesis and terpenoid skeleton biosynthesis. Therefore, these genes in related categories were discussed in detail. In our results, the candidate genes associated with starch and sugar metabolism that belong to the primary metabolic pathway were enriched via KEGG analysis. Starch that accumulates in potato tubers is a major source of carbohydrates and a primary storage compound, which is composed of amylopectin and amylose [56]. Starch and sucrose can be reversibly metabolized through coordinated enzymes in multiple pathways, including alpha-amylase (AMY), beta-amylase (BAM), starch phosphorylase (SP), ADP-glucose pyrophosphorylase (AGPase), starch synthases/granule-bound starch synthases (SS/GBSS), starch-branching enzyme (SBE) and starch debranching enzyme (DBE) for starch degradation and synthesis and sucrose-phosphate synthase (SPS), UDP-glucose pyrophosphorylase (UGPase), sucrose synthase (SuS), invertase (INV) and fructokinase (Frk) for sucrose synthesis and hydrolysis [57].

**Fig. 8:**
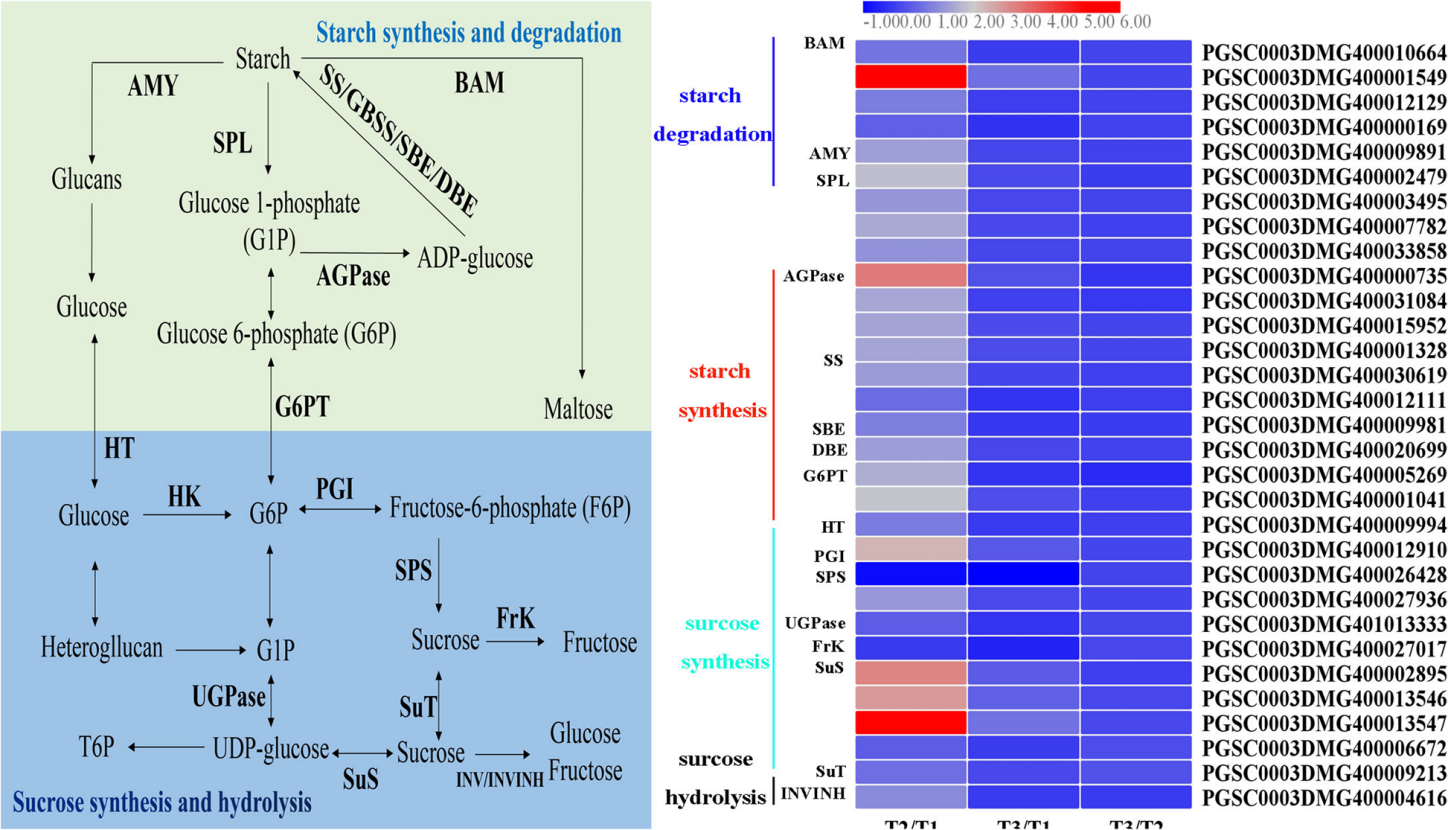
The starch and sugar metabolism pathway and the heatmap of DEGs involved in this pathway during wound healing of tubers (Each column represents the mean expression value (log_2_ FPKM, T2 sample are divided by T1, T3 sample are divided by T1 and T2, respectively) of three biological replicates obtained from RNA-Seq data)

AMY and BAM are the major enzymes that contribute to starch degradation. Four *BAM* genes were identified in our work, and only one *BAM* gene (PGSC0003DMG400001549) showed higher transcriptional levels in tissues including natural and BTH-induced healing. However, the transcript of the gene encoding amylase isoforms was observed to be upregulated in natural healing tissues but downregulated in BTH-induced healing tissues (Fig. 8). In the process of starch synthesis, AGPase plays a crucial role in catalysing the conversion of glucose-1-phosphate into the glucosyl donor ADP-glucose, which is then used to synthesize amylose by GBSS or amylopectin by SS, SBE and DBE [58]. Three *AGPase* genes were identified in this study, the expression pattern was the same as *BAM* and *AMY*. Only one *AGPase* gene was 3.2-fold more highly expressed by natural wound healing. Moreover, the *SS*, *SBE*, *DBE* and *G6PT* were also found only to be induced by natural wound healing according to the sequencing data.

SuS mainly catalyses the conversion between UDP-glucose and fructose to prepare substrates for starch synthesis. Four *SuS* genes were identified in this dataset, with PGSC0003DMG400002895 and PGSC0003DMG400013547 being the most abundantly upregulated with more than 3-fold in contrast with natural healing and non-healing, suggesting that they may be essential for starch synthesis. UDPase serves as a reversible enzyme for the interconversion of starch and sucrose and is also responsible for UDP-glucose production. Only one *UDPase* gene was identified in our results, and no response was displayed in different comparisons. SPS functions in the conversion of fructose into sucrose, and two *SPS* genes that were significantly downregulated in natural and BTH-treated healing tissues were annotated in our study. Another enzyme, FrK, converts fructose to fructose-6-phosphate, with a downregulation expression pattern in different tissues. Genes related to starch and sugar metabolism have been well researched in other species. However, the key genes and their regulatory mechanism in the process of wound healing remain unknown. The results presented herein may be helpful for further studying the molecular mechanism underlying starch and sucrose metabolism during wound healing of potato tubers.

#### Fatty acid metabolism

**Fig. 9:**
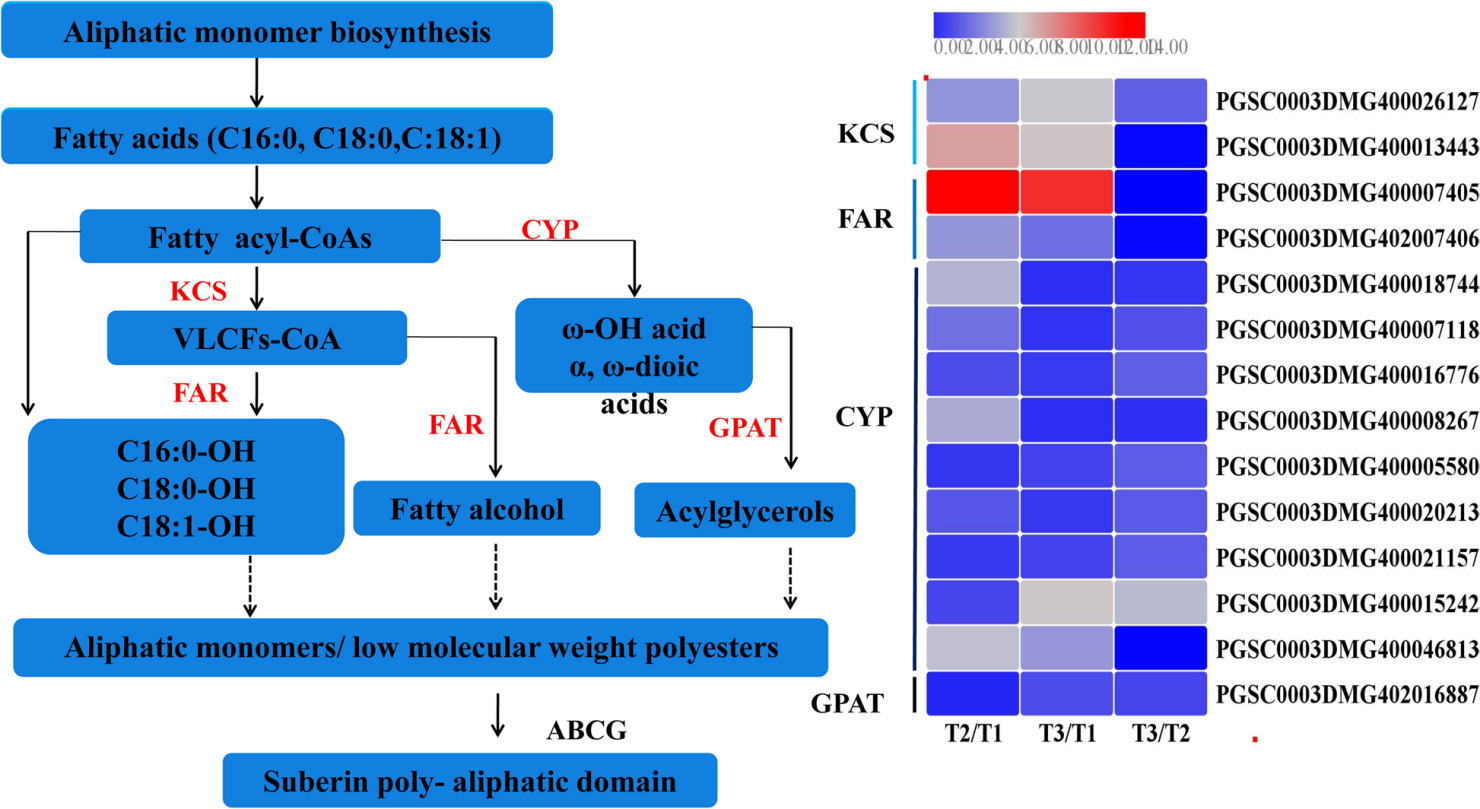
The fatty acid metabolism pathway and the DEGs involved in the pathway during wound healing of tubers (cytochrome oxidase, CYP; β-ketoacyl-CoA synthetase, KCS; Fatty acyl reductase, FAR. GPAT: Glycerol-3-phosphate acyltransferase. Each column represents the mean expression value (log_2_ FPKM, T2 sample are divided by T1, T3 sample are divided by T1 and T2, respectively) of three biological replicates obtained from RNA-Seq data)

Fatty acid metabolism yields a series of fatty acid-derived aliphatic constituents used for forming the suberin poly (aliphatic) domain (SPAD) [59]. KCS, CYP, FAR and GPAT serve as key enzymes in this metabolic pathway and are required for wound healing in potato tubers. β-Ketoacyl-CoA synthases (KCS) catalyse the production of very-long-chain fatty acids, and cytochrome P450 enzyme (CYP), a fatty acid ω-hydroxylase, is needed for ω-hydroxyacids and α,ω-diacids conversion [60]. Fatty acyl-CoA reductases (FARs) carry out the reduction of primary alkanols [61], and glycerol 3-phosphate acyltransferases (GPATs) catalyse monoacylglycerol formation [62]. Our transcriptomic analysis showed that a total of 14 genes related to fatty acid metabolism were differentially expressed under wounding healing, including 2 *KCS*, 2 *FAR*, 9 *CYP* and 1 *GPAT* (Fig. 9). Two *KCS* genes were upregulated significantly in T2/T1 and T3/T1 comparisons, the most upregulation fold of them reached 7.2-fold. *StKCS6* has been reported to involve in suberin and wax biosynthesis and scilencing of *StKCS6* leads to a reduction of the monomeric carbon chain lengths [63]. Two *FAR* genes identified in this study were all not expressed in non-healing tissues but showed significant upregulation pattern in response to natural and BTH-induced wound healing, the gene PGSC0003DMG400007405 in healing tissues was dramatically enhanced by 12.7- and 11.8 fold, respectively. In kiwifruit, FAR was documented to associate with primary alcohol formation in wound suberization [64]. As for *CYP* genes, most of them expressed at lower level in non-healing tissues but higher level in healing tissues, suggesting that CYP expression can be triggered by wound healing event. In potato, *StCYP86A33* is closely related with longer chain ω-OH fatty acid synthesis [65]. These results indicated that the genes related to fatty acid metabolism could be induced by wound healing and were regulated at the transcriptional level.

#### Phenylpropane biosynthesis

**Fig. 10:**
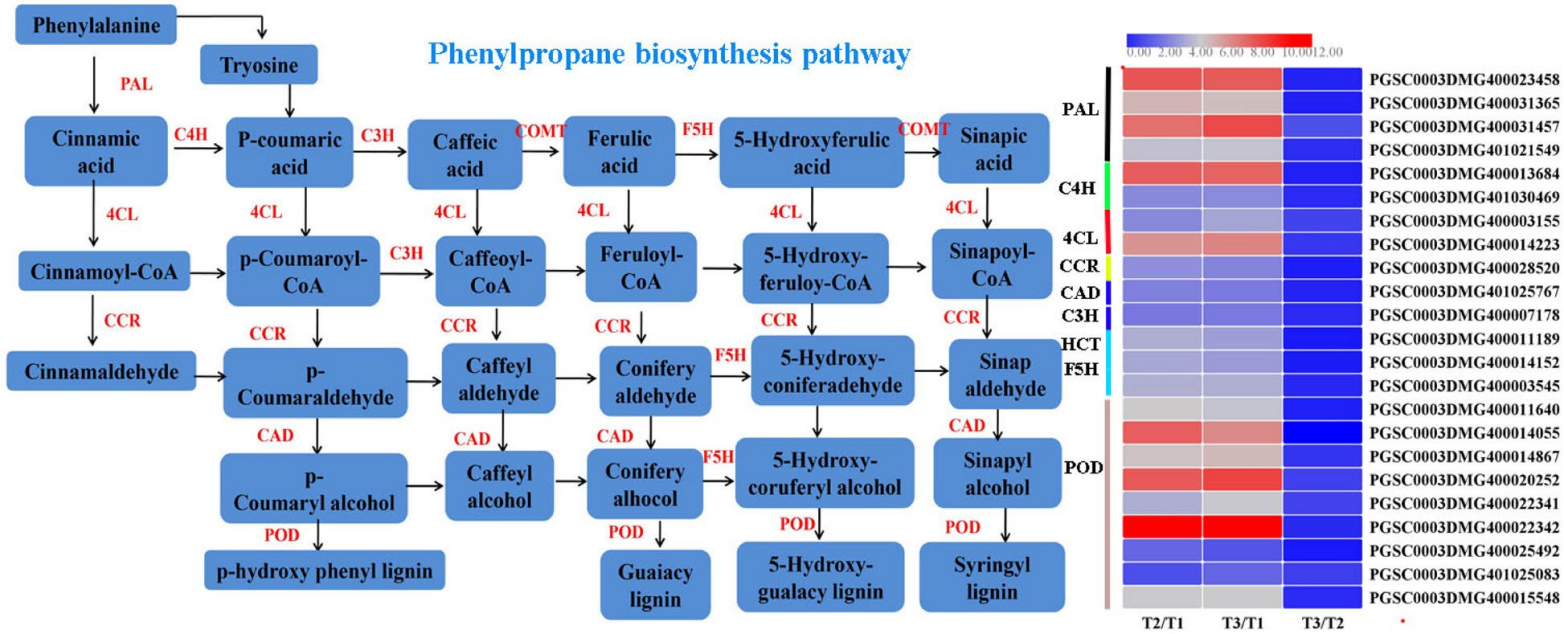
The phenylpropane biosynthesis pathway and the DEGs involved in this pathway during wound healing of potato tubers (phenylalanine ammonia-lyase, PAL; cinnamate-4-hydroxylase, C4H; 4-coumarate-CoA ligase, 4CL; Cinnamoyl-CoA reductase, CCR; cinnamyl alcohol dehydrogenase, CAD; Ferulic acid-5 coumaric acid CoA ligase, F5H; Peroxidase, POD; Each column represents the mean expression value (log_2_ FPKM, T2 sample are divided by T1, T3 sample are divided by T1 and T2, respectively) of three biological replicates obtained from RNA-Seq data)

The phenylpropanoid biosynthesis pathway plays an essential role in the process of wound healing in potato tubers, which provides the major precursors for the suberin polyphenol domain (SPPD). The key DEGs involved in this pathway included *StPAL*, *StC4H*, *St4CL*, *StCCR*, *StCAD*, *StF5H* and *StPOD *(Fig. 10). Phenylalanine ammonia lyase (PAL) is the first enzyme that converts phenylalanine into cinnamate, and cinnamic acid 4-hydroxylase (C4H) is responsible for yielding 4-hydroxycinnamic acid. 4-Coumarate-CoA ligase (4CL) functions in *p*-coumaric acid conversion in another phenylpropanoid route [66]. During wound healing, all of 4 *StPAL* homologues were significantly induced in the healing tissues. Two genes (PGSC0003DMG400023458 and PGSC0003DMG400031457) were upregulated the most in healing tubers, and the latter was enhanced by 7.5-fold and 8.6-fold in natural-healing and BTH-induced healing tubers, respectively. Both *StC4H* and* St4CL* in this pathway were enriched only two genes and were significantly upregulated in natural and BTH-treated wound healing. Additionally, the production of hydroxycinnamaldehydes depends on cinnamoyl-CoA reductase (CCR), and cinnamyl alcohol dehydrogenase (CAD) reduces hydroxycinnamaldehydes to their respective alcohols [67]. Regarding the expression of *StCCR*, *StCAD* and *StF5H* in our experiment, only one homologue of each gene was enriched and exhibited an upregulation change after wound healing, but their expression was not affected in BTH-induced healing vs. natural healing. The induced expression of these key genes by BTH was consistent to our previous finding on the promoted wound healing of potato tubers by elevating the phenylpropanoid metabolism [13].

Other biosynthetic enzymes in the phenylpropanoid biosynthesis pathway, such as quinate/shikimate hydroxycinnamoyl transferase (HCT) or *p*-coumaroylshikimate 3′-hydroxylase (C3′H), play an indispensable role in lignin biosynthesis [68, 69]. One homologue of *StC3H* (PGSC0003DMG400007178) and two *StHCT* homologues in this study were upregulated once tuber wound healing occurred. The POD gene family is extraordinarily large in potato. These genes are equipped with a variety of functions, including the oxidation of phenolic monomers in the formation of phenoxyl radicals [70]. In the present study, 9 *POD* genes enriched in this pathway were all upregulated in wound healing tissues under natural or BTH-treated conditions, two of which (PGSC0003DMG400022342, PGSC0003DMG400020252) were induced more than any others, the former was upregulated by 10.7 fold by wound healing. The above results suggested that these key genes might play a regulatory role in the phenylpropanoid biosynthesis pathway in response to natural or BTH-induced wound healing of potato tubers.

#### Terpenoid skeleton biosynthesis

**Fig. 11:**
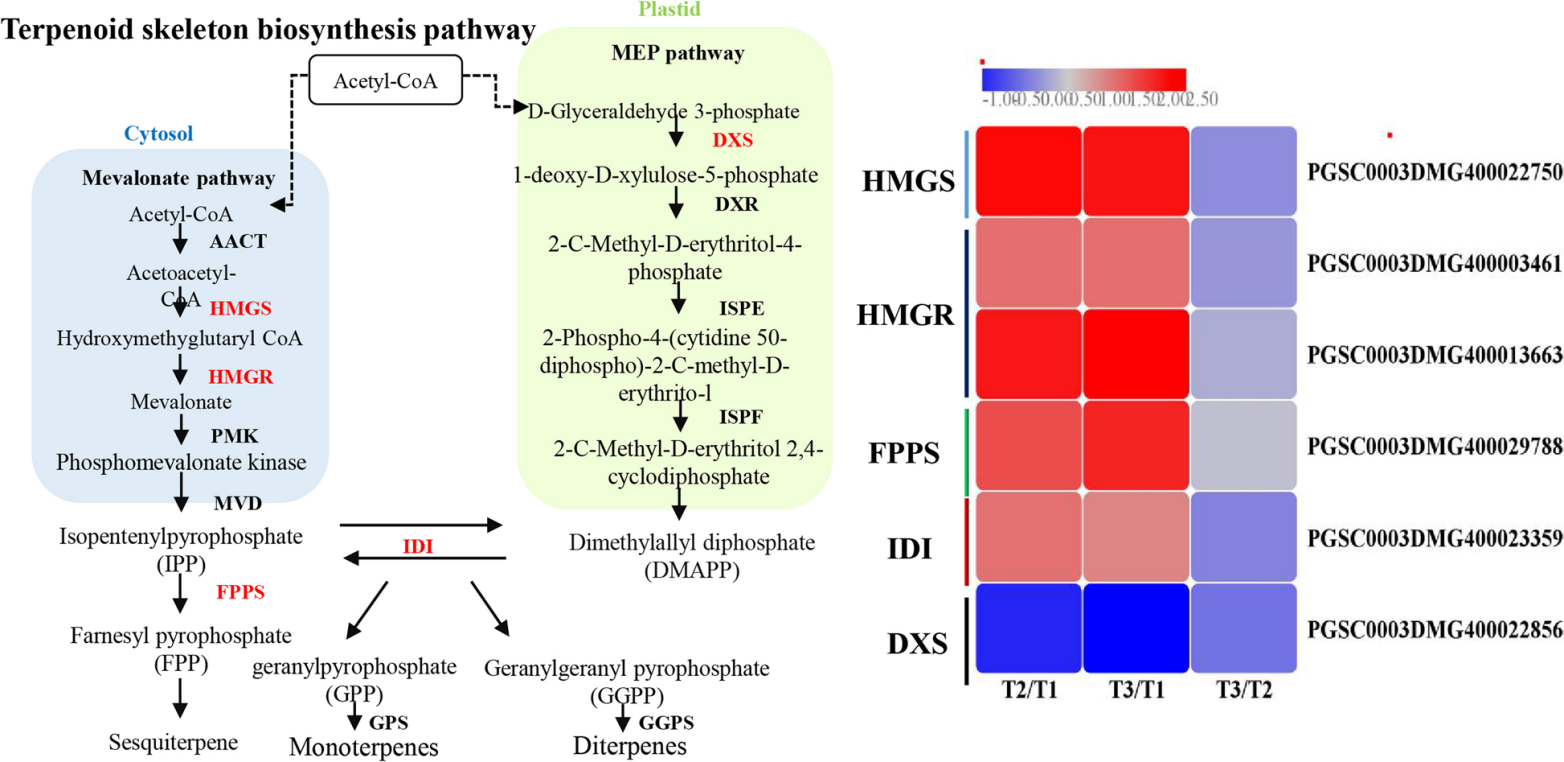
The terpenoid skeleton biosynthesis pathway and the DEGs involved in the pathway during wound healing of tubers (acetyl-CoA C-acetyltransferase, AACT; hydroxymethylglutaryl-CoA synthase, HMGS; hydroxymethylglutaryl-CoA reductase, HMGR; phosphomevalonate kinase, PMK; diphosphomevalonate decarboxylase, MVD; farnesyl diphosphate synthase, FPPS; isopentenyl-diphosphate Delta-isomerase, IDI; 1-deoxy-D-xylulose-5-phosphate synthase, DXS; 1-deoxy-Dxylulose-5-phosphate reductoisomerase, DXR; 4-diphosphocytidyl-2-C-methyl-D-erythritol kinase, ISPE; 2-C-methyl-D-erythritol 2,4-cyclodiphosphate synthase, ISPF; geranylgeranyl diphosphate synthase, GGPS. Each column represents the mean expression value (log_2_ FPKM, T2 sample are divided by T1, T3 sample are divided by T1 and T2, respectively) of three biological replicates obtained from RNA-Seq data)

There are various terpenoid compounds with diverse structures in plants that not only function in the process of plant-environment interactions but also play a role in plant growth and development [71]. These terpenoids are derived from two kinds of isomeric 5-carbon precursors called isopentenyl diphosphate (IPP) and dimethylallyl diphosphate (DMAPP), and the 2-C-methyl-D-erythritol 4-phosphate (MEP) and mevalonate acid (MVA) pathways are responsible for IPP and DMAPP synthesis [72, 73]. Under the action of prenyltransferases, these components are transformed into diphosphate (GPP), farnesyl pyrophosphate (FPP) and geranylgeranyl pyrophosphate (GGPP). The condensation of the C5 precursors leads to the formation of mono-, sesqui- and diterpenes [74]. Hydroxymethylglutaryl-CoA synthase (HMGS) and hydroxymethylglutaryl-CoA reductase (HMGR) are rate-limiting enzymes in the MVA pathway, which were identified to be homologous with known enzymes in this work and exhibited significantly induced expression levels in the wound healing tissues (Fig. 11). Farnesyl diphosphate synthase (FPPS) is responsible for the formation of farnesyl pyrophosphate (FPP) from IPP [75]. One gene encoding FPPS were identified from the transcriptomic data and were observed to be upregulated in both natural and BTH-induced healing tissues. The fundamental precursors IPP and DMAPP can be converted to each other by 1-deoxy-D-xylulose-5-phosphate synthase (IDI) [76], and the only gene encoding this enzyme was upregulated more1-fold in natural and BTH-treated healing tissues.

In contrast, another rate-limiting enzyme, 1-deoxy-D-xylulose-5-phosphate synthase (DXS), in the MEP pathway, which plays an important role in DMAPP formation, had a lower expression level and was downregulated in healing tissues. In addition, the downstream terpene synthase geranylgeranyl diphosphate synthase (GGPS) is used to catalyse geranylgeranyl pyrophosphate to synthesize diterpene [77]. According to the DEG annotation, the *GGPS* genes were detected to be expressed at very low level. These genes encoding *HMGS*, *HMGR*, *FPPS*,* IDI* and *DXS* were differentially expressed in the process of healing, suggesting that these steps may affect terpenoid synthesis. Pateraki and Kanellis reported that the rate-limiting enzymes CcHMGR and CcDXS in the terpenoid biosynthetic pathway showed an increased transcriptional level when the plant tissues (*Cistus creticus*) were mechanically wounded and treated with salicylic acid [78], which reflects the fact that this pathway was needed for wound stress. In addition, terpenoid release was reported to be capable of defending against pests [79] and pathogenic fungi [80]. Interestingly, except for steroids and carotenoids, a number of phytohormones, including cytokinins, abscisic acid and gibberellins, are derived from terpenoids [72, 81]. Therefore, characterizing these genes further provides a new understanding of the underlying action pattern and molecular mechanism of terpenoid biosynthesis during wound healing of potato tubers.

#### Verification of gene expression related to phenylpropane biosynthesis, fatty acid metabolism and plant-pathogen interactions

**Fig. 12:**
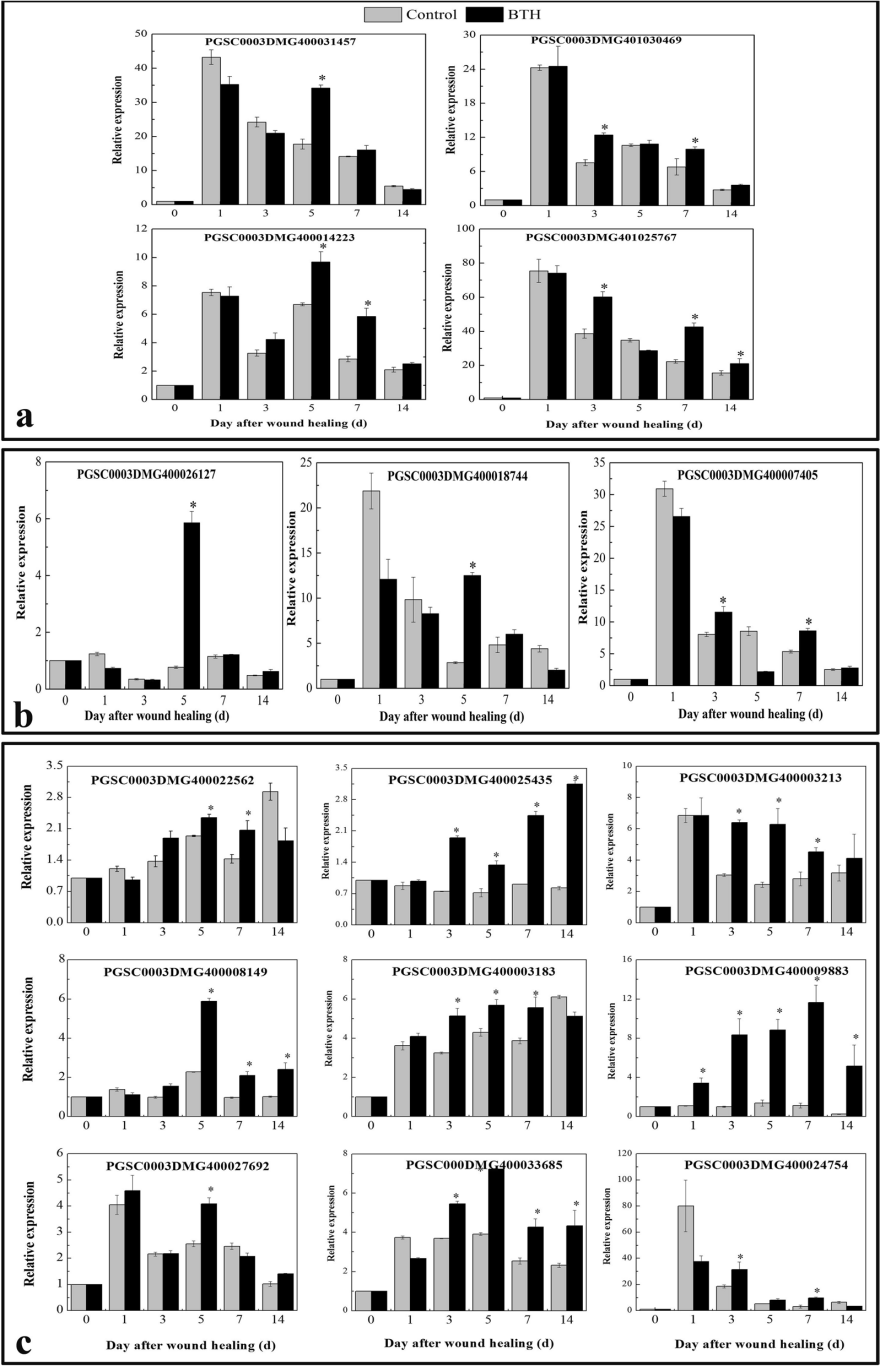
The relative expression level change of 16 selected genes (A-a: genes from phenylpropane biosynthesis, A-b: genes from fatty acid metabolism, A-c: genes from plant-pathogen interaction) of DEGs by qRT-PCR

To validate the expression profiles obtained from RNA-Seq analysis, 16 DEGs from three metabolic pathways were randomly selected to explore their expression profiles in wound healing tubers under BTH treatment using qRT–PCR. The *efla* gene was used as a control for expression normalization. These unigenes were involved in phenylpropane biosynthesis and fatty acid and plant-pathogen interaction pathways (Fig. 12). The following genes, *StPAL* (PGSC0003DMG400031457), *StC4H* (PGSC0003DMG401030469), *St4CL* (PGSC0003DMG400014223) and *StCAD* (PGSC0003DMG401025767), were induced by BTH at different time points and are involved in phenylpropane biosynthesis. The *StKCS* (PGSC0003DMG400026127) and *StCYP* (PGSC0003DMG400018744) genes only exhibited higher expression levels after BTH treatment at 5 days of healing, and another gene, StFAR (PGSC0003DMG400007405), was increased by BTH at 3 and 7 days of healing. However, most of the genes encoding Ca^2+^-binding proteins (PGSC0003DMG400022562/PGSC0003DMG400025435/PGSC0003DMG400003213/PGSC0003DMG400008149/PGSC0003DMG400013183/PGSC0003DMG400033685) involved in plant-pathogen interactions were significantly induced by BTH from 3 d to 14 d of healing.

**Fig. 13:**
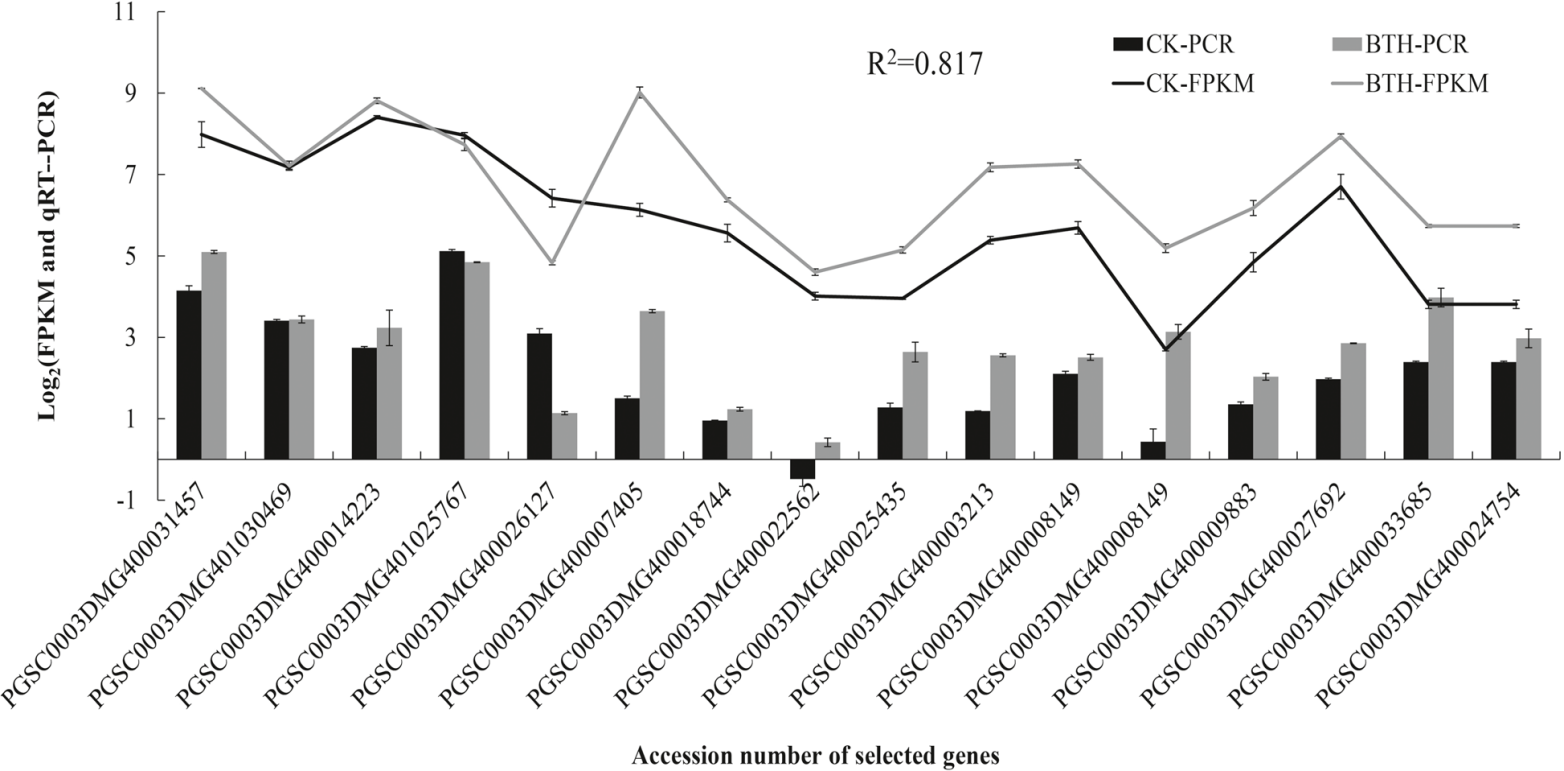
The relationship between RNA-Seq data (lines) and qRT-PCR data (columns). The correlation coefficient (*R*^2^) using log_2_ ratio measure of transcript levels of 16 genes is indicated in the figure

The results showed that although the expression patterns of the selected genes varied between RNA-Seq and qRT-PCR analysis, the trend of gene expression changes detected by qRT-PCR largely coincided with the transcriptome sequencing results (Fig. 13). Analysis of Pearson’s correlation coefficient showed that qRT-PCR detection for selected genes and RNA-Seq data at 5 d of healing were highly correlated. The correlation coefficient was 0.817, indicating that the RNA-Seq data were positively correlated with the qRT-PCR data, also indicating that the RNA-Seq data in this study are valuable.

## Conclusion

Our results provide the gene expression patterns related to wound healing of natural or BTH-treated in potato tubers, which also provides clues about important regulatory mechanisms, especially those involving plant hormones, transcription factors, Ca^2+^-mediated signal transduction, and a series of metabolic pathways, such as starch and sugar metabolism, fatty acid metabolism, phenylpropane biosynthesis, and terpenoid skeleton biosynthesis pathways, that participate in the healing process. The present research revealed the underlying mechanisms of natural or BTH-treated wound healing of potato tubers, which provided valuable information for understanding the transcriptional regulation processes of wound healing from another perspective.

## Methods

### Potato tubers and BTH treatment

Potato tubers ‘cv. Longshu No. 3’ free from physical injuries and infection was purchased at Gansu Ailan Potato Seed Industry Co. Ltd. and used immediately. The washing, surface sterilization and wounding treatment of tubers were performed as described by Jiang et al. [13]. Tubers were allowed to treat with sterile water and 100 mg/L BTH, which were subsequently stored at 20 °C and a relative humidity of 75–80% for wound healing. The healing tissues of the wounded surface at 0 d and 5 d were collected via manual dissection, frozen with liquid nitrogen, and archived at − 80 °C until further analysis.

### RNA extraction and RNA-Seq

Healing tissue sampling and RNA-Seq from three time points, 0 d-Control (T1), 5 d-Control (T2) and 5 d-BTH (T3), were carried out in parallel as three biological replicates. Samples were sent to Breeding in Xi’an, where the cDNA library was produced and sequenced using an Illumina HiSeq2500 system. To ensure that the bases of high quality were used for de novo assembly, the raw sequences were filtered by removing adaptor sequences and poor-quality reads and produced 64.49 Gb clean data in total, which were used for subsequent analysis, and the sequenced data were filtered by quality score (Q ≥ 30). The statistics of clean reads are listed in the Supplementary Table. S1. Then, all these clean reads were mapped to contig assemblies using TopHat 2 software and mapped to the reference genome of *Solanum tuberosum* downloaded from Potato Genomics Resource (PGSC, http://solanaceae.plantbiology.msu.edu/pgsc_download.shtml). The values of fragments per kilobase of transcript per million fragments mapped (FPKM) were calculated by cufflinks software; subsequently, DESeq software was used to analyse differentially expressed genes. Genes with |fold change (FC)| ≥ 2 and Q (false discovery rate of *P* values, FDR) < 0.01 were considered a threshold to judge the significance of differentially expressed genes.

### Functional annotation of genes

To understand the molecular mechanism, the predicted functions of all the unigenes were annotated based on a series of databases, such as NR (NCBI nonredundant protein sequences), Swiss-Prot, GO (Gene Ontology), COG (Clusters of Orthologous Groups of proteins), Pfam (Protein family), and KEGG (Kyoto Encyclopedia of Gene and Genome). Analysis of GO and KEGG pathway enrichment were used to investigate functions and pathways affected over wound healing progress. GO enrichment analysis was performed by Blast2GO software and KEGG enrichment [82] was performed by KOBAS 2.0 software.

### Differential expression verification by quantitative real-time PCR (qRT-PCR)

To validate the obtained results in the experiment, 16 differentially expressed genes were selected for qRT-PCR verification at 0, 1, 3, 5, 7 and 14 d. The accession numbers in the PGSC database and specific primers for each of these genes are presented in Supplementary Table S2. The RNA extraction kit (Cat. No. DP419, TIANGEN208 Biotech, China) was used to extract total RNA from fresh healing tissues (0.2 g), and then 1 μg of total RNA was reverse transcribed to generate first-strand cDNA using TIAN script RT Kit211 (Cat. No. KR116, TIANGEN Biotech, China). Quantitative RT-PCR was performed on a Light Cycler 96 SW 1.1 system with a volume of 20 μL reaction mix containing 1 μL cDNA, 10 μL 2× Super Real PreMix Plus with SYBR Green, 0.4 μL 50 × ROX Reference Dye, 0.6 μL forward and reverse primers and 7.4 μL RNase-Free ddH_2_O. Gene expression was normalized using elongation factor 1-alpha 1 (*ef1a*). PCR was run with the cycling parameters of 94 °C for 15 min, followed by 40 cycles at 95 °C for 30 s, 60 °C for 20 s and 72 °C for 30 s, and each sample was performed in triplicate. The 2^-△△^CT method was selected to calculate the relative fold change in template abundance for each sample [83].

### Statistical analysis

The statistical analysis was performed using Microsoft Excel 2007 and SPSS 19.0. Charts were drawn by OriginPro 8.1 software. The means among various groups were compared by Duncan’s multiple range tests. The data were analysed and are expressed as the means ± standard deviations (SDs), and *P* < 0.05 indicated significance differences.

## Supplementary Information

**Additional file 1.**

**Additional file 2.**

**Additional file 3.**

**Additional file 4.**

**Additional file 5.**

## Data Availability

All of data supporting our results are contained within the manuscript. All raw RNA-seq data in this manuscript are available for downloading from the NCBI Sequence Read Archive (BioProject ID: PRJNA757824).

## Abbreviations

DEGs: Differentially expressed genes
KEGG: Kyoto encyclopedia of genes and genomes
GO: Gene ontology
CDPK: Calcium-dependent protein kinase
qRT-PCR: Quantitative real-time PCR
RNA-seq: RNA sequencing
ABA: Abscisic acid
JA: Jasmonic acid
ROS: Reactive oxygen species
