# Supplementary material for: Transcriptome sequencing and differential expression analysis of natural and BTH-treated wound healing in potato tubers (Solanum tuberosum L.)
Source: BMC Genomics. 2022 Apr 5;23:263. doi: 10.1186/s12864-022-08480-1 (PMC8981635; doi:10.1186/s12864-022-08480-1)
Supplement: Supplementary file 1 — Additional file 1. [file 12864_2022_8480_MOESM1_ESM.docx]

| Samples | | Total Reads | Mapped Reads | Mapped Ratio | Uniq Mapped Reads | Uniq Mapped Ratio |
| --- | --- | --- | --- | --- | --- | --- |
| T1 | T1-1 | 57,153,758 | 52,170,698 | 91.28% | 43,553,165 | 76.20% |
|  | T1-2 | 48,738,580 | 44,769,582 | 91.86% | 35,451,377 | 72.74% |
|  | T1-3 | 56,652,256 | 51,771,888 | 91.39% | 43,342,041 | 76.51% |
| T2 | T2-1 | 54,595,668 | 46,904,482 | 85.91% | 38,499,535 | 70.52% |
|  | T2-2 | 60,976,020 | 53,077,552 | 87.05% | 43,039,787 | 70.58% |
|  | T2-3 | 49,293,742 | 42,921,196 | 87.07% | 35,111,320 | 71.23% |
| T3 | T3-1 | 55,065,568 | 46,650,342 | 84.72% | 38,390,943 | 69.72% |
|  | T3-2 | 72,704,104 | 62,085,424 | 85.39% | 47,740,365 | 65.66% |
|  | T3-3 | 60,703,836 | 50,981,694 | 83.98% | 41,938,554 | 69.09% |

Table. S1. The statistics of total reads and the mapped reads compared with the genome
