# Supplementary material for: Transcriptome sequencing and differential expression analysis of natural and BTH-treated wound healing in potato tubers (Solanum tuberosum L.)
Source: BMC Genomics. 2022 Apr 5;23:263. doi: 10.1186/s12864-022-08480-1 (PMC8981635; doi:10.1186/s12864-022-08480-1)
Supplement: Supplementary file 2 — Additional file 2. [file 12864_2022_8480_MOESM2_ESM.docx]

Table. S2. Primer sequences used for qRT-PCR analysis

| Genbank Accession No | Primer sequences(Forward) | Primer sequences(Reverse) | Product size (bp) | GC  (%) | Tm  (℃) |
| --- | --- | --- | --- | --- | --- |
| AB061263 | ATTGGAAACGGATATGCTCCA | TCCTTACCTGAACGCCTGTCA | 81 | 61/63 | 42/52 |
| PGSC0003DMG400031457 | GCGATGGCTTCTTACTGC | TCCTCCAAATGCCTCAAAT | 209 | 55/44 | 54 |
| PGSC0003DMG401030469 | CGCTCACTGGAAGAAACC | TCCTACCAACACCGAAAG | 113 | 55/50 | 52 |
| PGSC0003DMG400014223 | ACCGAAGCATAAAGTGAC | CCATAACCCTGACCAAGT | 192 | 44/50 | 49 |
| PGSC0003DMG401025767 | CACCAGAACAAGCAGCAC | GATGTCCCATTGCCTTTG | 156 | 55/50 | 52 |
| PGSC0003DMG400026127 | AAAGGTTCCGATGACAAG | CAAGAGGACCCAATGTAG | 142 | 45/50 | 50 |
| PGSC0003DMG400007405 | TTACTGGTGCTACTGGCTTCT | ACGTTGCATGGCTGACTT | 122 | 50 | 54 |
| PGSC0003DMG400018744 | TTACCAAACCTGCCTGAA | CTCTGTTTCTCCCTCACT | 127 | 45/50 | 50 |
| PGSC0003DMG400024754 | GATTCAGCGGTTCATTAG | TGTGATCCAAGCGTATTG | 187 | 44.4 | 49 |
| PGSC0003DMG400003213 | CGTTTCAGAGCCCGTATC | TGTCCCTCACCTTCACC | 202 | 53 | 55.6 |
| PGSC0003DMG400013183 | ATCACCCCTGGATTCAAAATGCT | CCATCAACATCAGCCAC | 256 | 49/52 | 45/50 |
| PGSC0003DMG400022562 | TGTGGAGTATTGGCGT | GCCTGAGCAGCAGTAAGT | 208 | 50 | 44/55 |
| PGSC0003DMG400025435 | CTACATCACCGCAGACG | TAGACCTTTCTTGCCACC | 178 | 50/52 | 55/50 |
| PGSC0003DMG400027692 | ACCTGCTTGGATTAGACGC | CAATTCCTTCGCCGATAT | 254 | 53/55 | 52/44 |
| PGSC0003DMG400033685 | TGAGGAGGAACTCAAAG | GCATCATACGGACAAACT | 194 | 47 | 44 |
| PGSC0003DMG400008149 | GCACCTGAAGAAGGCGTTTG | CCGTCCATCCTTGTCAGTGT | 157 | 60 | 55/55 |
| PGSC0003DMG400009883 | GGGCTGAGACTGAAAAGGGC | TGTTGGAGGGCTTCATCTGC | 163 | 60 | 60/55 |
